# Exploring Genotype-by-Environment Interactions of Chemical Composition of Raspberry by Using a Metabolomics Approach

**DOI:** 10.3390/metabo11080490

**Published:** 2021-07-28

**Authors:** Sara Durán-Soria, Delphine M. Pott, Frank Will, Jennifer Mesa-Marín, Mariusz Lewandowski, Karolina Celejewska, Agnieszka Masny, Edward Żurawicz, Nikki Jennings, Anita Sønsteby, Erika Krüger, Sonia Osorio

**Affiliations:** 1 Departamento de Biología Molecular y Bioquímica, Campus de Teatinos, Instituto de Hortofruticultura Subtropical y Mediterránea “La Mayora”, Universidad de Málaga-Consejo Superior de Investigaciones Científicas, Campus de Teatinos, 29071 Málaga, Spain; sarads@uma.es (S.D.-S.); dpott@uma.es (D.M.P.); jmesam@uma.es (J.M.-M.); 2 Institute of Beverage Research, Hochschule Geisenheim University, 65366 Geisenheim, Germany; Frank.Will@hs-gm.de; 3 The National Institute of Horticultural Research (INHORT), Konstytucji 3 Maja 1/3, 96-100 Skierniewice, Poland; mariusz.lewandowski@inhort.pl (M.L.); karolina.celejewska@inhort.pl (K.C.); agnieszka.masny@inhort.pl (A.M.); 4 Department of Genetics, James Hutton Institute, Invergowrie, Dundee DD2 5DA, UK; Nikki.Jennings@huttonltd.com; 5 NIBIO, Norwegian Institute of Bioeconomy Research, 1431 Ås, Norway; anita.sonsteby@nibio.no; 6 Institute of Pomology, Hochschule Geisenheim University, 65366 Geisenheim, Germany; Erika.Krueger@hs-gm.de

**Keywords:** berry, environment, fruit quality, nutritional value, flavor, anthocyanins, ellagitannins, volatiles

## Abstract

Promoting the consumption of fruits is a key objective of nutrition policy campaigns due to their associated health benefits. Raspberries are well appreciated for their remarkable flavor and nutritional value attributable to their antioxidant properties. Consequently, one of the objectives of present-day raspberry breeding programs is to improve the fruit’s sensory and nutritive characteristics. However, developing new genotypes with enhanced quality traits is a complex task due to the intricate impacts genetic and environmental factors have on these attributes, and the difficulty to phenotype them. We used a multi-platform metabolomic approach to compare flavor- and nutritional-related metabolite profiles of four raspberry cultivars (‘Glen Ample’, ‘Schönemann’, ‘Tulameen’ and ‘Veten’) grown in different European climates. Although the cultivars appear to be better adapted to high latitudes, for their content in soluble solids and acidity, multivariate statistical analyses allowed us to underscore important genotypic differences based on the profiles of important metabolites. ‘Schönemann’ and ‘Veten’ were characterized by high levels of anthocyanins and ellagitannins, respectively, ‘Tulameen’ by its acidity, and ‘Glen Ample’ for its content of sucrose and β-ionone, two main flavor contributors. Our results confirmed the value of metabolomic-driven approaches, which may foster the development of cultivars with enhanced health properties and flavor.

## 1. Introduction

Red raspberry (*Rubus idaeus* L.) is one of the most important berry fruit crops whose consumption has increased dramatically over the last decade [1]. This increased demand is mainly due to the fruits’ well-known benefits for human health [2], but also because they are highly valued for their delicate flavor and appearance [3]. Fruit appearance is caused by multiple traits, but mainly relies on firmness, shelf life, and external characteristics, such as color and size. Health benefits rely on the composition of secondary metabolites, in particular, polyphenols, like anthocyanins and ellagitannins [4], while the overall flavor is mainly due to content of sugars, acids, and volatile compounds [5]. Polyphenols are shown to have potent antioxidant, anti-microbial, and anti-inflammatory effects [6], are responsible for fruit color, and also participate in the sensory attributes [7].

Similarly to many other important crops, the raspberry plant has received an intensive breeding focus over the last decades, where yield, fruit size, and harvesting traits have been targets, resulting in indirect reductions in flavor (aroma, taste, and texture) and nutritional compounds [8]. Nowadays, breeding is more focused on taste quality and an improved nutritional value, which require better understanding of the genetic control of the metabolic pathways which regulate metabolite abundance [9]. Up to now, combining metabolomics with two major approaches, (i) quantitative trait locus (QTL) analysis that usually focuses on bi-parental populations, and (ii) genome-wide association studies (GWAS), has proven to be a powerful tool to dissect complex metabolic traits and aid in crop improvement through maker-assisted selection [10,11,12,13,14,15,16,17]. In this regard, metabolic profiling coupled with the QTL mapping approach has been successfully carried out to identify different regulatory and structural genes involved in the control of metabolite level in raspberry fruits for volatile organic compounds, anthocyanins, and antioxidant capacity [18,19,20,21,22].

There are several studies that have shown that flavor and nutritional value are highly influenced by both endogenous factors (genotype) and environmental factors (latitudes, weather conditions, and postharvest practices) [23,24,25,26,27,28,29]. For instance, in strawberries, it has been described that post- and pre-harvest treatments with UV-C light improve fruit quality, increasing the levels of sucrose, vitamin C, and ellagic acid [30,31], while visible light spectrum treatment is shown to affect flavonoid accumulation, particularly anthocyanins [32,33]. Furthermore, different cultural systems, including compost socks and conventional and organic agricultural practices, have also been found to affect fruit quality, in terms of healthier and tastier metabolites [24,27,34,35].

Metabolomics can provide a snapshot of dynamic biological processes and provide a more holistic view of metabolic network behavior. Metabolomics can also complement the regulatory characterization of metabolic processes in a comparative analysis of cultivars grown under contrasting environments. Multivariate statistical analysis of these datasets can provide a more detailed view of the relationships between biological components that show differential behavior under contrasting environments in a species. In the present work, our primary objectives were to explore metabolic biodiversity and provide detailed comparative analyses of primary and secondary metabolism in ripe fruits of four raspberry cultivars (‘Glen Ample’, ‘Schönemann’, ‘Veten’ and ‘Tulameen’). Cultivars were chosen for their popularity; in particular, ‘Glen Ample’ and ‘Tulameen’ are important for European markets (‘Glen Ample’ in Scotland, Norway, and Germany and ‘Tulameen’ in Germany). In addition, due to their different origins (Supplementary Table S1), they show different adaptations to environmental conditions, allowing us to compare how primary and secondary fruit metabolism are modulated when these raspberry cultivars were grown in different European climates under similar agricultural practices. For future perspectives, this study can serve as a reference for further works that aim to correlate metabolites to various fruit-quality traits, facilitating the breeding of superior raspberry genotypes.

## 2. Results

### 2.1. Environment Data

Four commercial raspberry cultivars (cv. ‘Glen Ample’, ‘Schönemann’, ‘Tulameen’, and ‘Veten’) were cultivated in soil at three different locations in Germany, Poland, and Norway. Fully ripe fruits were collected for quality parameters and metabolome analysis on two harvest dates.

Table 1 presents information about environmental factors, such as mean temperature, mean radiation, and total precipitation, of the different growing locations in 2018. The sites were located at a distance of 11 degrees in latitude, influencing day-length, and thus flowering and harvest dates. Mean temperature of the different locations was lower with higher latitude, and in addition it is worth noting that total precipitation was significantly higher in Poland during the summer (harvest season).

### 2.2. Quality Attributes Are Impacted by Both Genetic and Environmental Factors

**Figure 1:**
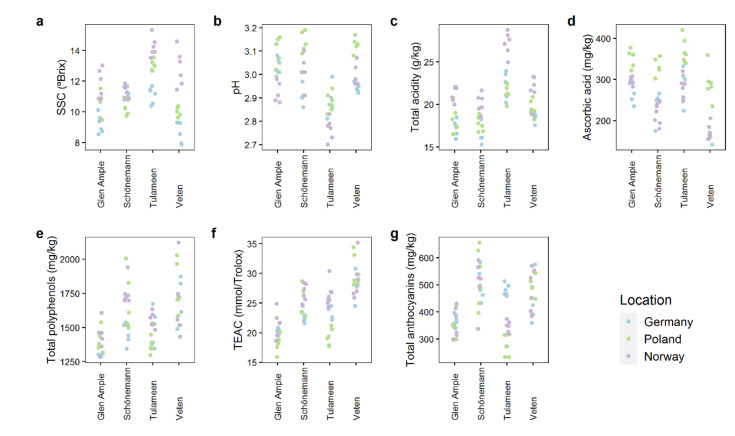
Soluble solid content (SSC) (**a**), pH (**b**), total acidity (**c**), ascorbic acid content (**d**), total polyphenols (**e**), Trolox equivalent antioxidant capacity (**f**), and total anthocyanins (**g**) of the fruits of the different raspberry genotypes. Each dot represents an individual sample, with color denoting the harvest location. The data represent two independent harvests.

Selected quality attributes were measured to assess fruit differences between cultivars grown in the three locations (Figure 1). Interestingly, soluble solid content (SSC) and total acidity were highest in all cultivars at the Norwegian location, with a mean value of 12.56° and 22.56 g/kg in Norway for SSC and acidity, respectively, compared to 10.01° and 18.78 g/kg in Germany and 11.03° and 19.16 g/kg in Poland. (Figure 1a–c). Curiously, the SSC content in ‘Schönemann’ appeared to be more stable across the different environments, displaying a lower range of variation in °Brix values (9.74–11.86°), while ‘Veten’ values exhibited extensive changes (from 7.86° in Germany to 14.6° in Norway). ‘Tulameen’ showed more differences in fruit acidity compared to the other genotypes, having the highest total acidity (23.49 g/kg, while the other cultivars displayed acidity values < 20 g/kg) (Figure 1c) and, consequently, the lowest pH (Figure 1b), in addition to a higher ascorbic acid concentration (314.72 mg/kg, while the other cultivars displayed ascorbic acid concentration < 304 mg/kg) (Figure 1d). Interestingly, ascorbic acid concentration was highest in Poland for all the genotypes (Figure 1d). The results obtained in total polyphenols and total anthocyanins are quite similar: ‘Glen Ample’ (1386 and 356 mg/kg, respectively) and ‘Tulameen’ (1476 and 366 mg/kg, respectively) had significantly lower concentration than did ‘Schönemann’ (1627 and 511 mg/kg, respectively) and ‘Veten’ (1706 and 473 mg/kg) (Figure 1e,g). Moreover, Trolox Equivalent Antioxidant Capacity (TEAC) partially matches total polyphenol and anthocyanin profiles, with ‘Veten’ (29.17 mmol/Trolox) showing the highest antioxidant capacity, followed by ‘Tulameen’ (23.32 mmol/Trolox) and ‘Schönemann’ (24.74 mmol/Trolox), which do not present perceivable differences, and finally by ‘Glen Ample’ (19.97 mmol/Trolox) with the lowest antioxidant capacity (Figure 1f). Regarding anthocyanins, polyphenols, and antioxidant capacity, ‘Glen Ample’ presented similar lower concentrations across all locations, unlike the other cultivars (Figure 1e–g). Noteworthily, ‘Tulameen’ displayed a significant increase in total anthocyanins in the German location, with a mean concentration of 480 mg/kg compared to 299 and 346 mg/kg in Poland and Norway, respectively (Figure 1g).

### 2.3. Metabolomic Analyses

In order to obtain a more detailed overview of the compounds responsible for the raspberry fruit differences, we performed metabolomic analyses of primary metabolites and secondary metabolites and volatiles.

Metabolite profiling of primary metabolites was obtained by using gas chromatography time-of-flight mass spectrometry (GC–TOF-MS) to identify 47 primary metabolites, which included 18 amino acids, 14 sugars and sugar derivates, 13 organic acids, and 2 polyamines (Supplementary Table S2). Secondary metabolite profiling was performed by high-performance liquid chromatography-mass spectrometry (HPLC-MS) and reverse-phase HPLC coupled to ultraviolet detector (RP-HPLC/UV) to identify 25 secondary metabolites, including eight ellagitannins, seven anthocyanins, six flavonols, two phenolic acids, one flavan-3-ol, and one condensed tannin (Supplementary Table S3). Automated headspace solid-phase microextraction (HS-SPME) sampling coupled to GC–MS allowed us to detect of 65 volatiles, grouped into 25 esters, 15 aldehydes, 10 ketones, 6 terpenes, 6 alcohols, 2 furans, and 1 alkane (Supplementary Table S4).

**Figure 2:**
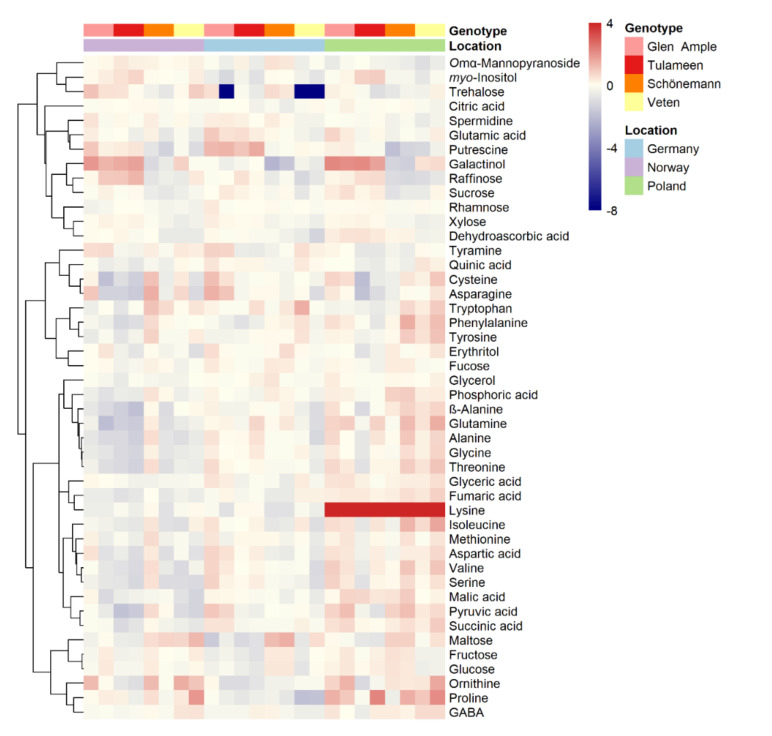
Heatmap visualization of raspberry primary metabolites identified in 2018 in two different harvests. Each value represents the normalized (median-centered and log_10_-transformed) mean of three biological replicates, with red and blue colors denoting relatively high and low intensities. Metabolites are clustered based upon Pearson correlation coefficients. Samples’ location and genotype are indicated with color labelling.

**Figure 3:**
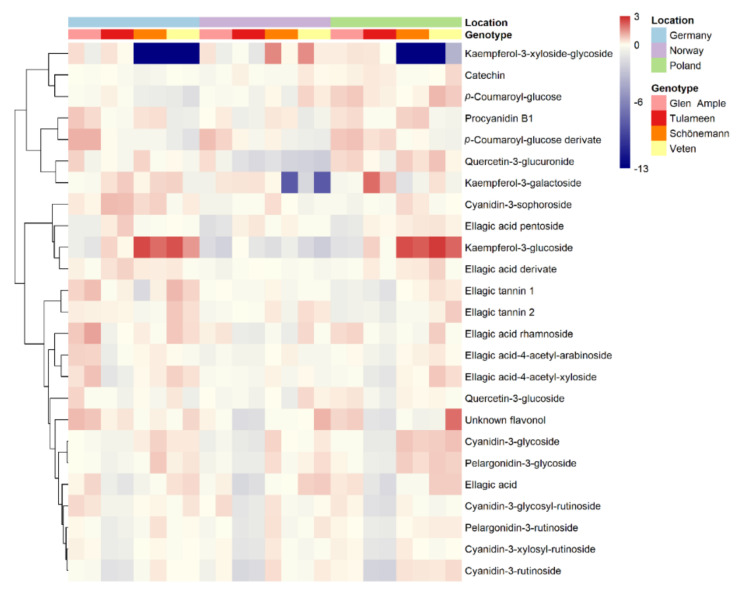
Heatmap visualization of raspberry secondary metabolites identified in 2018 in two different harvests. Each value represents the normalized (median-centered and log_10_-transformed) mean of three biological replicates, with red and blue colors denoting relatively high and low intensities. Metabolites are clustered based upon Pearson correlation coefficients. Samples’ location and genotype are indicated with color labelling.

**Figure 4:**
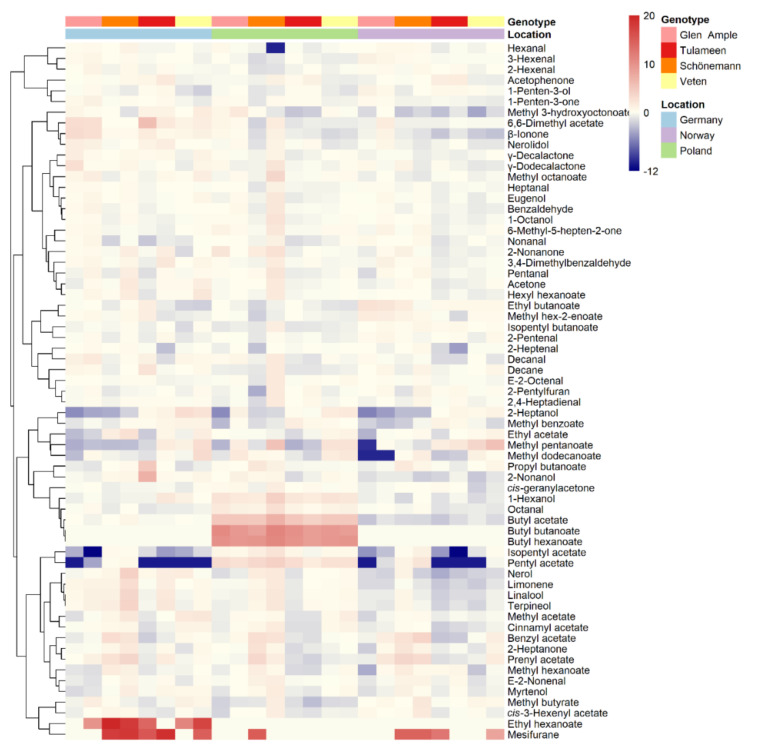
Heatmap visualization of raspberry volatile compounds identified in 2018 in two different harvests. Each value represents the normalized (median-centered and log_10_-transformed) mean of three biological replicates, with red and blue colors denoting relatively high and low intensities. Metabolites are clustered based upon Pearson correlation coefficients. Samples’ location and genotype are indicated with color labelling.

To obtain a better view of the fruit metabolic profiles, all metabolites are shown in heatmap representations (Figure 2, Figure 3 and Figure 4), where metabolites are clustered by a Pearson’s correlation based on their mean relative accumulation value in mature raspberries and outlining relationships within fruit metabolism. Metabolic patterns can be observed based on location, more obvious in primary metabolites and volatiles (Figure 2 and Figure 4), and on genotype, more visible in secondary metabolism (Figure 3). 

**Figure 5:**
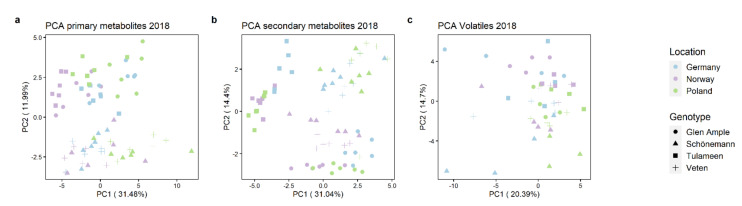
Principal component analysis (PCA) plots showing distributions for primary metabolites (**a**), secondary metabolites (**b**) and volatiles (**c**) of raspberry fruits. Each dot represents an individual sample, with shapes indicating the different raspberry cultivars, and colors the different locations. PC1 and PC2 represent the first and the second principal component, respectively.

Unsupervised principal component analyses (PCA) were performed on all datasets, revealing that both environmental and genetic factors influence primary and secondary metabolites and volatile content (Figure 5). It is difficult to discern if primary metabolite separation is more influenced by the genotype or environment; however, ‘Veten’ and ‘Schönemann’ group together, with principal component (PC) 2 separating them from ‘Glen Ample’ and ‘Tulameen’ and explaining 11.99% of the variation (Figure 5a). In the case of secondary metabolites, genotype clearly has a stronger impact in sample distribution than does the environment, with a clear separation of ‘Tulameen’ from the remaining cultivars based on PC1 (31.04% of variation). Interestingly, the separation between ‘Schönemann’ and ‘Veten’ is not so apparent with secondary metabolites, as was noted in their primary counterparts (Figure 5b). In contrast, patterning based on volatile compound profiles seems to be more affected by the location, even if a perceptible separation of ‘Schönemann’ from the remaining cultivars is observed (Figure 5c).

The effect of genotype on fruit metabolic composition, with volatiles showing a greater influence of growing conditions on their final content than for primary metabolites and most undoubtedly secondary metabolites, was confirmed by broad-sense heritability (H^2^) values (Supplementary Table S5). H^2^ is the proportion of a phenotypic trait explained by the overall variance for the genotype [36]. Interestingly, secondary metabolites showed higher H^2^ values compared to primary metabolites and volatile compounds, with more than half of them (15 of 25) displaying high H^2^ values (H^2^ > 0.4), while only 9 of 47 primary metabolites and 8 of 65 volatiles, respectively, showed H^2^ > 0.4 (Supplementary Table S5).

### 2.4. Key Metabolites for Fruit Quality Attributes Differentiate the Assessed Cultivars

Next, sparse partial least squares-discriminant analysis (sPLS-DA) was conducted on primary and secondary metabolites and volatile profiles to (1) separate the different genotypes based on metabolic traits and (2) to determine which compounds contributed the most to cultivar discrimination. Indeed, sPLS-DA is a supervised method, which focuses on class separation and additionally allows variable selection [37].

Three components were necessary for the separation of the four assessed cultivars based on their primary and secondary metabolite and volatile contents (Supplementary Figure S1). As expected, sPLS-DA analysis for primary and secondary metabolites confirmed the tendency observed in PCA analyses, highlighting a close relationship between ‘Schönemann’ and ‘Veten’ regarding metabolic composition (Supplementary Figure S1a,b).

Variable importance in projection (VIP) scores were calculated for each metabolite to determine the main compounds contributing to sample separation in the sPLS-DA plots. Metabolites with VIP values ≥ 1 were considered important for genotype separation and are shown in Supplementary Figure S2.

Interestingly, major discriminating primary metabolites included sugars, with xylose and sucrose showing the highest levels in ‘Glen Ample’, raffinose and galactinol showing increased levels in ‘Tulameen’, and fucose highest in ‘Schönemann’. Other primary metabolites with VIP scores ≥ 1 comprise putrescine, tyramine and dehydroascorbic acid (increased in ‘Glen Ample’), phosphoric acid (increased in ‘Schönemann’), and the aromatic amino acids phenylalanine and tyrosine (both increased in ‘Veten’) (Supplementary Figure S2). It is worth noting that the most discriminating metabolites also showed higher H^2^ values (Supplementary Table S5).

**Figure 6:**
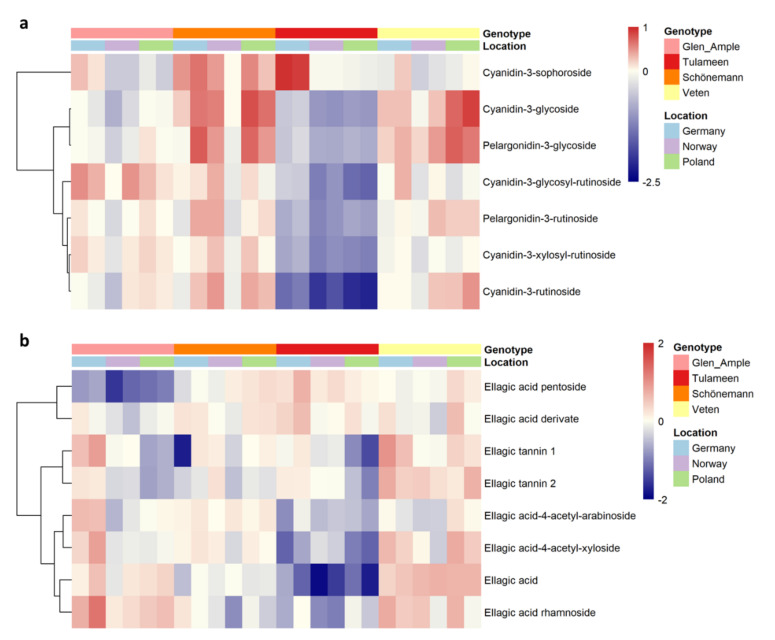
Heatmap visualization of raspberry anthocyanins (**a**) and ellagitannins (**b**) identified in 2018 in two different harvests. Each value represents the normalized (median-centered and log_10_-transformd) mean of three biological replicates, with red and blue colors denoting relatively high and low intensities. Metabolites are clustered based upon Pearson correlation coefficients. Samples’ location and genotype are indicated with color labelling.

Regarding secondary metabolite profiles, the distribution of anthocyanin and ellagic acid derivatives, the two main groups of secondary metabolites, appears to be cultivar-specific. Interestingly, ‘Tulameen’, together with ‘Glen Ample’, shows lower total polyphenol content and anthocyanin content (Figure 1 and Figure 6). Taking a deeper look into the anthocyanin profiles (Figure 6a), it is worth noting that the lowest content of all pigment derivatives was in ‘Tulameen’, with the notable exception of cyanidin-3-sophoroside, which displayed particularly high levels in the German location (Figure 6a). Anthocyanin levels were higher in ‘Schönemann’, in particular, cyanidin-3-rutinoside, -3-glycoside, -3-xylosyl-rutinoside and pelargonidin-3-glycoside, and -3-rutinoside, being some of the most discriminative metabolites in the sPLS-DA analysis with VIP scores > 1 (Supplementary Figure S2). On the contrary, ‘Veten’ is characterized by a high content of ellagic acid derivatives (Figure 6b), with ellagic acid as the secondary metabolite showing the highest VIP score. Procyanidin B1 and *p*-coumaroyl derivate, both with higher levels in ‘Glen Ample’, and ellagic acid pentoside, increased in ‘Tulameen’, are also important for cultivar discrimination (Supplementary Figure S2).

Discriminative volatiles (VIP ≥ 1) with enhanced content in ‘Schönemann’ include a series of acetate esters (prenyl, benzyl, cis-3-hexenyl, isopentyl acetates), methyl hexanoate, E-2-nonenal, 2-heptanone, hexanal and terpenoid volatiles (myrtenol, linalool, nerol, nerolidol, and terpineol). Other important discriminative volatiles comprise acetophenone, methyl benzoate and 1-penten-3-ol (higher in ‘Tulameen’), 1-penten-3-one (higher in ‘Glen Ample’), 2-heptanol and the acetate esters methyl, ethyl, and cinnamyl acetates (higher in ‘Veten’) (Supplementary Figure S2). Noteworthily, the apocarotenoid β-ionone, an important contributor to the raspberry floral aroma [38], also participates in the separation of ‘Glen Ample’ from ‘Veten’ and ‘Tulameen’; however, with VIP < 1 being highest in the first cultivar (Supplementary Figure S2).

### 2.5. Environment Impact on Important Aroma Volatiles

**Figure 7:**
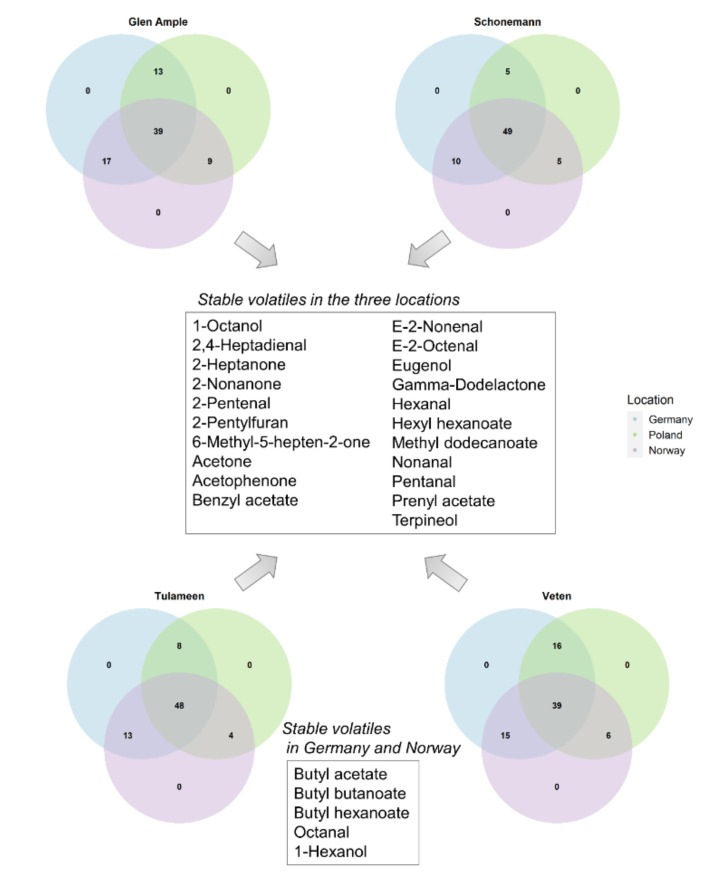
Venn diagrams showing stable volatiles (*p* < 0.05, ANOVA) among the different locations in the four assessed cultivars. Volatiles with stable content in the three locations (central box) and in both the Germany and Norway growing sites (lower box) are listed. Only the volatiles for which content did not differ significantly among locations in the four cultivars independently are listed.

Volatile content is strongly affected by the environment, as shown by PCA analyses and H^2^ values (Figure 5, Supplementary Table S5). ANOVAs were run for each cultivar to determine which metabolites were stably accumulated in the three locations, or in at least two of them (Figure 7; Supplementary Table S6). Interestingly, 21 metabolites did not show any significant differences between locations in any of the four assessed genotypes, including aldehydes and ketones (Figure 7).

Curiously, Poland was characterized by particularly high levels of some butyl esters in the four assessed cultivars, i.e., butyl acetate, hexanoate and butanoate, with the latter two exclusively detected in this location (Figure 4 and Figure 7). In 2019 (data not shown), butyl acetate was also strikingly increased in Poland compared to Germany and Norway; however, neither butyl butanoate nor butyl hexanoate could be surely detected. Octanal and 1-hexanol were also significantly increased in the Polish location in the four genotypes (Supplementary Table S4; Figure 7). Pentyl acetate was also increased in Poland; however, not significantly. Interestingly, fruits of ‘Glen Ample’ and ‘Schönemann’ sampled in Germany showed higher contents of important contributors to raspberry aroma, i.e., terpenoid volatiles, including β-ionone, limonene, nerolidol, linalool, and nerol; however, this increased content was only detected in one of the harvest dates. Additionally, ethyl hexanoate was strongly higher in the German location for ‘Schönemann’ and ‘Veten’ cultivars (Supplementary Table S4).

## 3. Discussion

Raspberry fruit metabolic composition and quality traits are influenced both by genetic and environmental factors, as observed in this and previous analyses [25,27,39]. While the present knowledge about the complex genetic architecture of fruit quality attributes has been extensively improved since the development of QTL mapping and GWAS approaches, combined with high throughput metabolomic platforms [40], the impact of the environment on fruit metabolite composition is still poorly understood, its analysis being additionally hampered by current unstable climatic conditions triggered by climate change [41,42,43,44,45]. Furthermore, the metabolic characterization of raspberry genotypes for sensory and nutritional qualities remains essential for fruit breeding strategies.

### 3.1. Temperature, Radiation and Precipitation Impact Key Quality Attributes

Important differences were observed in the metabolism of fruits grown in Norway, Poland, and Germany. Primary metabolism seems to be particularly affected, as quality attributes such as SSC, total acidity, and ascorbic acid content, which mainly rely on sugar and organic acid levels, clearly showed differences in the four assessed cultivars, depending on the growing site. SSC is an estimation of fruit sugar content, although other abundant compounds, such as organic acids, also influence its value. In this sense, the higher SSC observed in the four cultivars in the Norwegian location could be, at least partially, explained by their higher organic acid levels, and thus total acidity. Citric acid content, the main acid in raspberry fruits, together with fructose and glucose levels, two of the predominant sugars, were higher in fruits harvested in Poland and Norway (the two coldest locations) and may in part be responsible for higher SSC and acidity. Notably, concentrations of fructose, glucose, and citric acid have been reported to be negatively affected by increased temperatures in blackcurrant (*Ribes nigrum*), strawberry (*Fragaria* x *ananassa*) and grape (*Vitis vinifera*) fruits [46,47,48,49]. Additionally, dry content matter was significantly higher in the Norwegian samples (17.4% as a mean value among the assessed cultivars) compared to the other two locations (14.6% in Poland and 13.9% in Germany), suggesting that a dilution effect may differentially impact SSC and total acidity depending on the cultivation site. Together, our results imply that higher latitudes may be more favorable for the accumulation of important sensory attributes (dry matter, SSCs and acidity), as previously described in strawberries [50], which definitely could impact raspberry quality. Furthermore, high-latitude summers are characterized by long-day photoperiods; ‘Glen Ample’ raspberries grown under controlled environments in long-day conditions showed enhanced concentrations of organic acids, such as malic and quinic acids, compared to short-day photoperiods [51]. However, it is worth noting that both ascorbic acid and its oxidized form (dehydroascorbic acid), the two redox states of vitamin C, are significantly increased in the samples harvested in Poland, the location which was characterized by unusually high precipitations during the harvest season (summer). Environmental influence on vitamin C content has been previously described [52,53], with precipitation during summer favoring its accumulation in blackcurrant fruits [54].

Furthermore, secondary metabolites, including polyphenols and volatile compounds, play decisive functions in mediating plants’ responses towards their environment [55]. In this sense, an impact on both their content and profiles can be expected to change depending on the cultivation site, latitude, and harvest date. For example, both anthocyanin accumulation and composition are strongly influenced by abiotic factors, such as light and temperature [39,56], with cyanidin glucosides reported to be UV-absorbing pigments [18,19]. Notably, levels of cyanidin-3-sophoroside, the main anthocyanin present in ripe raspberries [57], were increased in the four cultivars harvested in Germany, the location with the higher mean temperature and radiation during spring–summer 2018; this increase was especially remarkable for ‘Tulameen’. Additionally, levels of cyanidin- and pelargonidin-3-glycosides tended to be lower in the Norway-harvested samples, possibly as a consequence of the lower temperature and sunlight. Lower latitudes within Europe have also shown to favor anthocyanin accumulation in strawberries and currants (*Ribes* sp.) compared to higher ones, with significant differences in pigment pattern between southern and northern samples [58,59,60,61,62]. However, total phenols and strawberry fruit antioxidant capacity were generally increased in higher latitudes, which may be partially explained by higher ascorbic acid and tannins; curiously, our data showed a trend of increased TEAC values (mean TEAC value of 25.88 mmol/Trolox in Norway, compared to 23.8 and 23.19 mmol/Trolox in Germany and Poland, respectively) in the Norwegian samples, except for ‘Veten’. None of the measured tannins (procyanidin B1 and ellagitannin derivatives) showed increased content in the Norway-harvested fruits; however, a deeper profiling of this class of polyphenols might be necessary to identify the metabolites responsible for this high antioxidant capacity in higher latitudes.

The raspberry fruit volatilome is dominated by terpenes, apocarotenoids, acids, alcohols, and esters [63]. In particular, the carotenoid-derived β-ionone was characterized as the most important aroma contributor, conferring floral notes to raspberry’s scent [38,64]. Interestingly, our data indicate that β-ionone levels were increased in the German cultivation site in the four cultivars, being significant for both harvest dates in ‘Glen Ample’ and ‘Tulameen’ genotypes. β-ionone is derived from the enzymatic cleavage of carotenoids, pigments involved in plant photoprotection and acclimation to light exposure [65]. Carotenoid increase in light-exposed grapes led to enhanced apocarotenoid-derived volatiles in the ripe fruits [66,67], suggesting that the higher β-ionone content detected in the German-harvested raspberries may be a consequence of elevated radiation, as compared to Norwegian and Polish growing sites.

### 3.2. Polyphenol Content Is Highly Genotype-Dependent

The raspberry fruit is a particularly rich source of polyphenols, with anthocyanins and ellagitannins being the most abundant classes [20,27]. Curiously, the two assessed cultivars with higher polyphenol content, ‘Schönemann’ and ‘Veten’, show contrasting profiles between both classes, with ‘Veten’ presenting a high content of ellagic acid, a metabolite originating from the natural hydrolysis of ellagitannins known for its strong antioxidant properties, converting it into a powerful protective substance against various neurodegenerative and inflammatory diseases [68,69]. In contrast, ‘Schönemann’ was characterized by elevated levels of cyanidin and pelargonidin derivatives in all locations and harvests (responsible for its dark-red color), except for the second harvest date in Norway [70]. Remarkably, while both cultivars show similar levels of total polyphenols (1627 and 1706 mg/kg for ‘Schönemann’ and ‘Veten’, respectively), ‘Veten’ is notable for its higher antioxidant capacity (24.74 and 29.17 mmol/Trolox for ‘Schönemann’ and ‘Veten’, respectively). These data confirmed previous analyses which demonstrated that ellagitannins and derivatives were the main contributors to raspberry antioxidant capacity [20,71,72,73]. Total anthocyanins and, to a lesser extent, ascorbic acid content have also been described as contributing to raspberry antioxidant capacity [39,74,75], and may explain the higher TEAC value measured in ‘Tulameen’ fruits (23.32 mmol/Trolox) compared to ‘Glen Ample’ (19.97 mmol/Trolox). ‘Glen Ample’ was previously described as a low-polyphenol (including low anthocyanins, resulting in light-red fruits), low-ascorbic acid and low-SSC genotype in a study comparing ten cultivars grown in a Norwegian location [5,25]. On the other hand, ‘Veten’ was characterized by even lower ascorbic acid levels, concomitant with our results, but high concentrations of polyphenols and anthocyanins (conferring it a dark-violet color), converting it in one of the preferred cultivars for processing in Norway [25]. Furthermore, high levels of ellagitannin derivatives in ‘Veten’ may also influence its sensory quality due to their astringency [20,76].

### 3.3. Flavor-Related Metabolites May Explain Differential Sensory Perception among Raspberry Cultivars

Although our results outlined that both primary metabolites and volatile compounds were more strongly affected by the environment than secondary metabolites (polyphenols), sPLS-DA analyses allowed us to highlight a few metabolites important for genotype discrimination and for fruit sensory perception. ‘Tulameen’ presents the highest total acidity (>20 g/kg), with values coinciding with a previous study [5]. Interestingly, the same authors reported that ‘Tulameen’ might be perceived as too acidic and astringent, based on a sensory analysis. On the other hand, sucrose, of which the highest levels in ‘Glen Ample’ were found to be positively correlated with sweetness, is a key parameter for raspberry overall appreciation score [77]. ‘Glen Ample’ is the predominant cultivar grown in Norway due to its marketability (good appearance, high yield and firmness) [78], and even if our study suggests that its sucrose content, together with its higher levels of aroma-impacting β-ionone, may positively influence sensory perception, Aaby et al. [5] concluded that it could be negatively perceived due to its sour odor and green flavor.

‘Schönemann’ was notable for its atypical volatile profile, compared to the other three genotypes. In particular, it showed elevated monoterpene (linalool, terpineol, and nerol) and acetate ester content, although this increase was strongly affected by the growing site. Interestingly, a sensory analysis of four raspberry cultivars identified ‘Schönemann’ for its atypical/strange and musty odor [70], suggesting that its singular volatile profile has a negative impact on its aroma perception. In particular, its higher content of monoterpene may be responsible for its musty notes, as described in mandarins (*Citrus reticulata*) [79].

## 4. Material and Methods

### 4.1. Plant Material and Fruit Yield

‘Glen Ample’ was developed by the James Hutton Institute (Scotland); ‘Schönemann’ is an old German cultivar; ‘Veten’ comes from Norway; and ‘Tulameen’ is from Canada. It is worth mentioning than ‘Schönemann’ and ‘Veten’ share ‘Lloyd George’ as a parent, and ‘Glen Ample’ and ‘Tulameen’ share ‘Glen Prosen’ in their pedigree. (Supplementary Table S1).

Plants of the four raspberry cultivars, ‘Glen Ample’, ‘Schönemann’, ‘Tulameen’, and ‘Veten’, were grown and harvested at the Hochschule Geisenheim University (HGU) in Geisenheim, Germany (49°59’ N, 7°58′ E), Norwegian Institute of Bioeconomy Research (NIBIO) in Kapp, Norway (60°40′ N, 10°87′ E), and Instytut Ogrodnictwa (INHORT) in Skierniewice, Poland (51°91′ N, 20°05′ E), under similar agricultural practices defined by a pre-agreed growing protocol.

Approximately 500 g of fully mature fruits were collected at two different harvest dates during June–July 2018 in Germany and Poland and during July–August 2018 in Norway, being immediately frozen in liquid nitrogen. Frozen samples of each cultivar were separated in six biological replicates—three per harvest date—and ground into a powder using a TissueLyser II (Qiagen) and stored at −80 °C until analysis.

### 4.2. SSC, pH, Total Acidity, Ascorbic Acid and TEAC Measurements

Soluble solid content (SSC, in °Brix) was evaluated with a refractometer by adding a few drops onto the lens. pH was determined with 5 g of raspberry puree in 25 mL milli-Q-water with a pH-meter. Total titratable acidity was determined by diluting 5 g of raspberry puree in 25 mL milli-Q-water, adding 0.1 M NaOH to an end pH of 8.1. Results were referred to as grams of citric acid per 100 g of fresh weight. For each biological replicate, depending on the fruit size, a pool of 25–30 fruits was used.

The Trolox equivalent antioxidant capacity (TEAC) assays, measuring the ability of antioxidant molecules to quench the ABTS·+ radical cation (2,2′-azinobis (3-eth-ylbenzothiazoline-6-sulfonate) in comparison with Trolox standards, were performed as previously described [80,81] on 0.32 mg of frozen sample. 10 μL of extract and 1000 μL of radical reagent were incubated together, and the absorbance at 734 nm was measured in a spectrophotometer after 6 min. Results were expressed in μmoles of Trolox equivalents per gram of fresh weight (μmol TE/100 g FW).

### 4.3. Metabolite Profile Analysis

Primary metabolite extraction, derivatization and analysis by GC–TOF-MS was performed as previously reported [82]. The obtained mass spectra were cross-referenced with the Golm Metabolome database [83].

Secondary metabolite analysis by HPLC coupled to ion trap mass spectrophotometer (anthocyanins) and RP-HPLC/UV (colorless polyphenols) was carried out as described in [59]. Volatile analysis by HS-SPME/GC-MS was executed following the protocol presented by Pott et al. [84] All metabolite-relative content obtained from the three techniques was normalized to dry weight.

### 4.4. Statistical Analysis

All data were relativized to a control sample. R software was used for multivariate statistical analysis (https://www.R-project.org/). We used pheatmap R package (https://CRAN.R-project.org/package = pheatmap, accessed on 25 May 2021) to determine metabolic up- and down-regulation in a heatmap representation, performed on the sample mean values of the three biological replicates. Each value was median-centered, log10-transformed, and clustered based upon Pearson correlation coefficients [14].

Principal component analysis (PCA) was applied using unit variance scaling, and sparse partial least squares discriminant analysis (sPLS-DA) was performed with the mixOmics R package [37]. The variable importance in projection (VIP) scores were then applied to select the discriminant metabolites among genotypes [85].

Stable volatiles in two or three of the assessed locations were represented in a Venn diagram with the VennDiagram R package (https://CRAN.R-project.org/package = VennDiagram, accessed on 21 May 2021), employing one-way analysis of variance (ANOVA) for each genotype separately, with a statistical significance of *p* ≤ 0.05. Broad sense heritability (H^2^ = V_G_/V_P_; V_G_ being the total genetic variance and V_P_ the total phenotypic variance) was calculated from the variance components obtained by ANOVA [86].

## 5. Conclusions

By using a combination of metabolomic tools, we performed an extensive characterization of four raspberry cultivars grown in three distinct locations in Europe. Multivariate statistical analyses allowed us to outline the genetic and environmental factors controlling the accumulation of quality-related metabolites, both for the organoleptic and nutritional characteristics of this highly appreciated fruit. The cultivars evaluated here appear to be better adapted to north-Europe growing conditions (Norwegian location), based on their dry matter, soluble solid, and acidity contents. Furthermore, differential metabolite patterns in the four assessed cultivars could be correlated with previous sensory studies, suggesting that metabolomic approaches are suitable for assessing fruit quality. This study may pave the way for the development of future European-bred cultivars, which will combine high levels of taste- and aroma-related compounds with elevated anthocyanin and ellagitannin concentrations. It is therefore expected that this information, combined with breeding strategies, will allow the establishment of elite cultivars with increased commercial value, and adapted to local growing conditions, directly impacting the competitiveness of the food industry sector by achieving a more efficient production.

## Figures and Tables

**Table 1:** Climatic factors at the locations where fruits were harvested. Values are placed chronologically according to raspberry growth and reproduction period, from March to June 2018. NIBIO: Norwegian Institute of Bioeconomy Research; INHORT: The National Institute of Horticultural Research, HGU: Hochschule Geisenheim University.

Location	Month	Mean Temperature (°C)	Mean Radiation (W/m^2^)	Total Precipitation (mm^3^)
NIBIO (Norway)	March	−5.4	2365.6	17.5
	April	5.6	4251.9	3.0
	May	8.5	4200.7	91.4
	June	14.4	4678.7	103.6
	July	20.7	6233.9	27.0
	August	14.7	3783.2	59.0
INHORT (Poland)	March	−1.6	2281.3	13.2
	April	13.2	4842.3	28.6
	May	16.5	6092.0	51.6
	June	18.5	5880.7	30.0
	July	20.3	5467.4	155.0
HGU (Germany)	March	4.8	2341.3	38.7
	April	14.0	4705.9	19.4
	May	17.5	5982.0	71.6
	June	20.0	6013.3	74.8
	July	21.3	6085.0	41.9

## Data Availability

Data is contained within the article or supplementary material.

## Supplementary Materials

The following are available online at https://www.mdpi.com/article/10.3390/metabo11080490/s1, Figure S1: Sparse partial least square discriminant analysis (sPLS-DA) score plot for primary metabolites (a), secondary metabolites (b) and volatiles (c), where each dot represents an individual sample, and each color and shape indicate the different raspberry cultivars grown in 2018 in the two different harvests, Figure S2: VIP scores for primary, secondary metabolites and volatiles from sPLS-DA analyses. Only metabolites with VIP ≥ 1 are shown, Table S1: Information about raspberry genotypes used in this study, Tables S2 Table S3 Table S4: Primary, secondary metabolites and volatile datasets, Table S5: Broad-sense heritability (H^2^) for primary, secondary metabolites and volatile compounds, Table S6: Common volatiles between locations in the four assessed genotypes.
