# Supplementary material for: Exploring Genotype-by-Environment Interactions of Chemical Composition of Raspberry by Using a Metabolomics Approach
Source: Metabolites. 2021 Jul 28;11(8):490. doi: 10.3390/metabo11080490 (PMC8398420; doi:10.3390/metabo11080490)
Supplement: Supplementary file 1 [file metabolites-11-00490-s001.zip › supplementary figures.pdf]

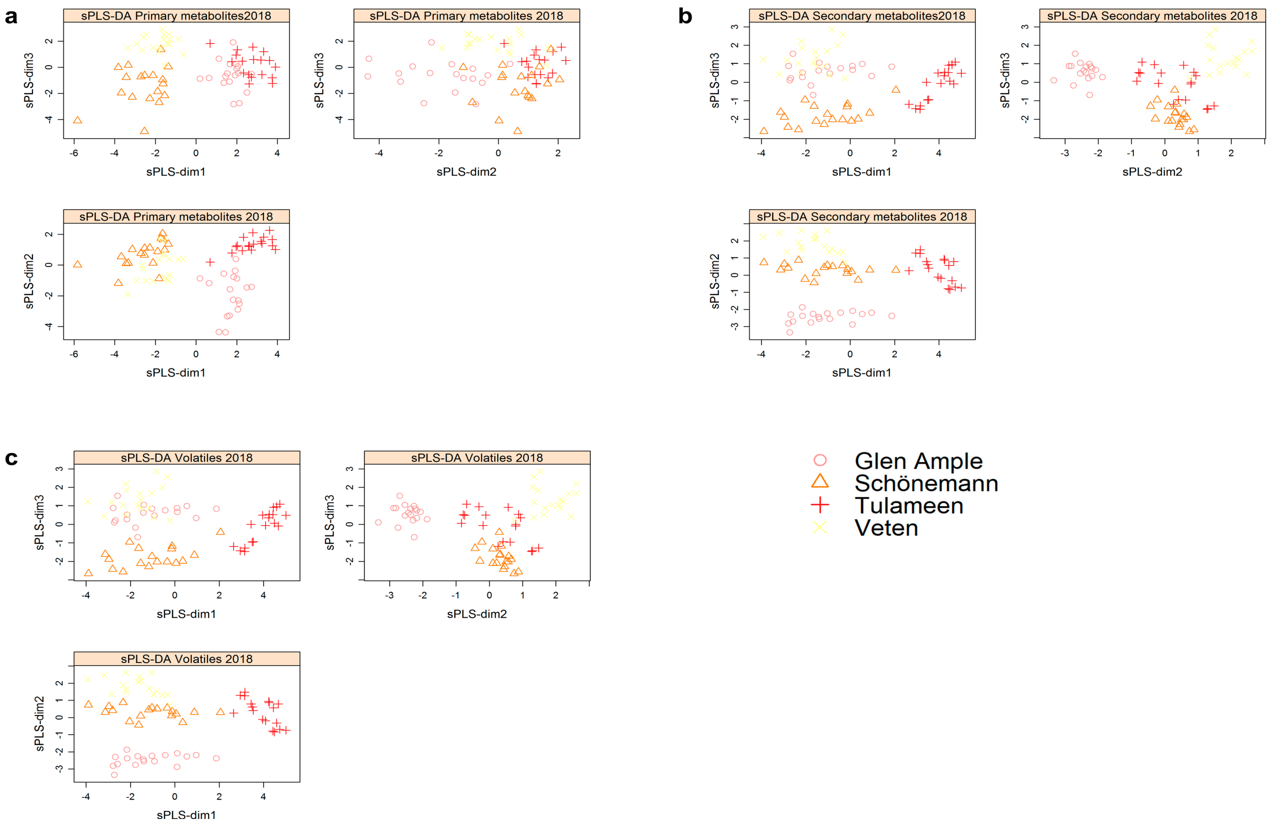

Figure S1. Sparse partial least square discriminant analysis (sPLS-DA) score plot for primary metabolites (**a**), secondary metabolites (**b**) and volatiles (**c**), where each dot represents an individual sample, and each color and shape indicate the different raspberry cultivars grown in 2018 in two different time harvests.

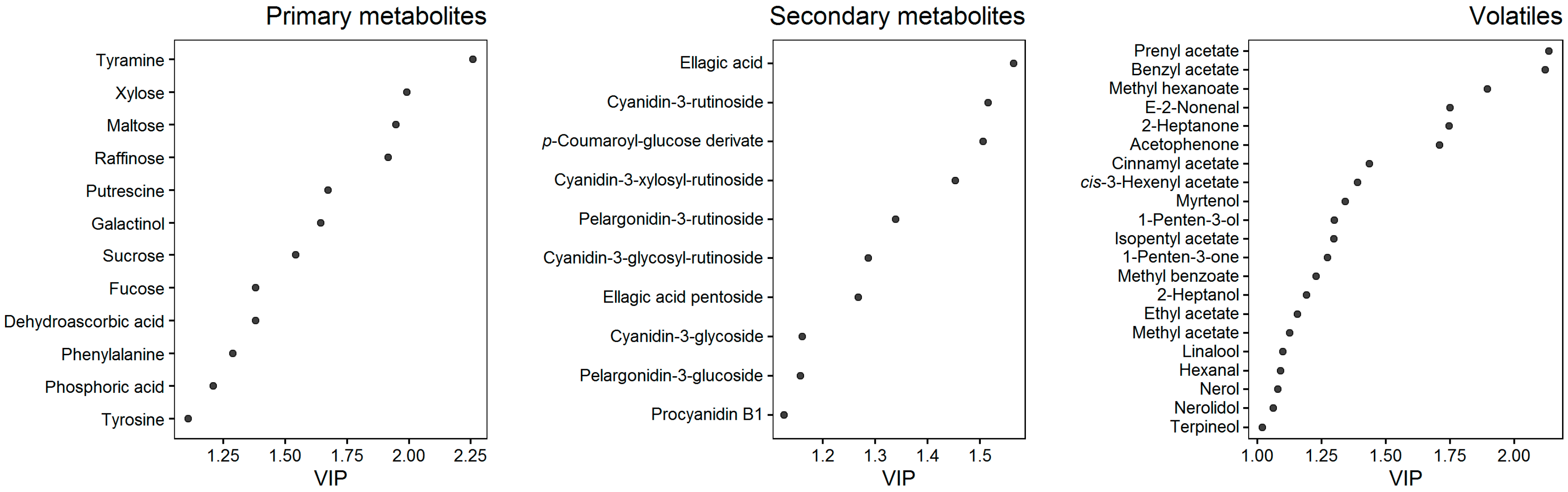

Figure S2. VIP scores for primary. secondary metabolites and volatiles from sPLS-DA analyses. Only metabolites with VIP ≥ 1 are shown.
